# New Molecular Technologies for Minimal Residual Disease Evaluation in B-Cell Lymphoid Malignancies

**DOI:** 10.3390/jcm7090288

**Published:** 2018-09-18

**Authors:** Irene Dogliotti, Daniela Drandi, Elisa Genuardi, Simone Ferrero

**Affiliations:** 1 Hematology Division, Department of Molecular Biotechnologies and Health Sciences, University of Torino, 10123 Torino, Italy; irenedogl@hotmail.com (I.D.); daniela.drandi@unito.it (D.D.); elisa.genuardi@unito.it (E.G.); 2 Division of Hematology 1U, Azienda Ospedaliero Universitaria Città della Salute e della Scienza, 10126 Torino, Italy

**Keywords:** minimal residual disease, PCR, next generation sequencing, droplet digital PCR, circulating tumor DNA, lymphoma, leukemia, myeloma, lymphoproliferative diseases

## Abstract

The clearance of malignant clonal cells significantly correlates with clinical outcomes in many hematologic malignancies. Accurate and high throughput tools for minimal residual disease (MRD) detection are needed to overcome some drawbacks of standard molecular techniques; such novel tools have allowed for higher sensitivity analyses and more precise stratification of patients, based on molecular response to therapy. In this review, we depict the recently introduced digital PCR and next-generation sequencing technologies, describing their current application for MRD monitoring in lymphoproliferative disorders. Moreover, we illustrate the feasibility of these new technologies to test less invasive and more patient-friendly tissues sources, such as “liquid biopsy”.

## 1. Introduction

### 1.1. The Early Steps and Prognostic Importance of Minimal Residual Disease

During recent decades, a large number of studies have shown that minimal residual disease (MRD) detection significantly correlates with clinical outcomes in many hematologic malignancies. Different molecular technologies, mostly polymerase chain reaction (PCR) based methods, have been studied for MRD evaluation in lymphoid disorders. Since the discovery of PCR [1], tools have been rapidly evolving. Notably, the first study describing the clinical usefulness of PCR for MRD detection in lymphoma was published by Gribben JG, in 1991 [2]. A while later the real time TaqMan based PCR (qPCR) was introduced, allowing for quantification of molecular targets [3]. Presently, qPCR is the most validated and standardized method for MRD detection, even if some drawbacks are still not overcome [3,4,5,6].

While the above-mentioned molecular methods are currently widely employed for MRD monitoring in lymphoma and in acute lymphoblastic leukemia (ALL), other methods, such as multicolor flow cytometry (MFC), have major role for MRD detection in chronic lymphocytic leukemia (CLL) and multiple myeloma (MM) [7,8,9].

MRD information by molecular methods is nowadays considered crucial for clinical decision-making in ALL. In fact, it has been described that detection of MRD during the initial phases of therapy, in combination with other relevant clinical and biological characteristics, allows for correct patients’ risk groups stratification [10]. Based on these evidences, MRD monitoring has been implemented also in lymphoma patients, for which it is currently under evaluation as a prognostic and treatment-guiding tool. For instance, MRD is now employed in the context of follicular lymphoma (FL) and mantle cell lymphoma (MCL) studies (EudraCT numbers: 2012-003170-60, 2012-001676 and NCT02354313), sponsored by Fondazione Italiana Linfomi (FIL, see www.filinf.it) [11].

From a technical point of view, several PCR tools are available, using different principles and approaches. Strategies used for PCR-based MRD can be classified into two subgroups: qualitative and quantitative approaches. Qualitative strategies usually consist of nested or semi-nested PCR assays, with a sensitivity of 1.00 × 10^−5^; however, they give limited information due to the lack of target quantification [12]. Several groups have developed quantitative real-time PCR (qPCR) methods, that enable accurate assessment of the number of residual tumor cells at consecutive follow-up time points [3,4,5,6,13,14,15].

In this review, we present the basic concepts that underlie the new generation’s tools lately introduced within the panorama of molecular methods for MRD monitoring in lymphoproliferative disorders.

### 1.2. MRD Potential Molecular Targets in B-Cell Hematologic Malignancies

An ideal molecular marker is present only in neoplastic cells; therefore, the target choice for MRD monitoring strongly depends on the disease subtype. During early B-cell differentiation, rearrangement of the different segments of the immunoglobulin (IG) genes occurs. Each neoplastic lymphoid cell harbors identically rearranged IG heavy chain (IGH) genes, making them the ideal tumor-specific marker for MRD monitoring in B-cell lymphoprolifarative disorders [16]. In addition, qPCR assays detecting incomplete, non-functional IGH rearrangements and immunoglobulin light chains (IGκ, IGλ) have been attempted [14,15]. However, these last molecular targets are not currently employed in MRD monitoring, due to lower sensitivity thresholds (1.00 × 10^−4^) [17].

Moreover, disease-specific chromosome translocations can also be employed as MRD PCR targets [18]. Actually, *t* (14;18) occurs in about 90% of patients with FL, and involves the antiapoptotic BCL2 and IGH genes. BCL2/IGH translocation is considered to be the founding event of FL lymphomagenesis. In addition, *t* (11;14), occurring in >95% of MCL, juxtaposes the BCL1 to the IGH gene, resulting in the overexpression of CCND1 which enables malignant transformation by deregulating cell cycle [18].

Finally, single-nucleotide mutations can also be exploited for molecular monitoring of specific diseases.

Recently, the MYD88^L265P^ point mutation was found as highly recurrent (90%) in Waldenström Macroglobulinemia (WM), prompting the development of ASO (allele-specific oligonucleotides) PCR assays for both mutational screening and quantification of MYD88^L265P^; other, less frequent mutations in WM include CXCR4 and ARID1A, detected in 23% and 17% of patients, respectively [19,20,21]. Moreover, B-RAF^V600E^ mutation was described as virtually present in 100% of hairy cell leukemia (HCL) patients [22], being a useful diagnostic and therapeutic target.

However, not every B-cell lymphoid disorder is characterized by reliable disease-specific molecular alterations applicable to all patients for MRD purposes. In Hodgkin lymphoma (HL), in fact, identification of specific genetic features for disease monitoring is still challenging, since malignant cells are usually very rare in bulk tissue (<5%), though some recurrent aberrations have been identified (e.g., IGH rearrangements, mutations in TNFAIP3, XPO1, NFκB, JAK/STAT genes) [23,24]. Similarly, diffuse large B cell lymphoma (DLBCL) is a molecularly heterogeneous neoplasia so marker screening needs to be performed, on each individual patient, to determine the mutational pattern to use for MRD application [25].

### 1.3. qPCR-MRD Approaches: Technical Considerations and Feasibility on Different Tissues

The sensitivity of a qPCR approach depends on several factors, including the type of rearrangement, size of the junctional region and amount of DNA available for each reaction. MRD level can be reliably quantified in the majority of cases in which a relatively high number of pathological cells is present, while if their level is very low, the assay becomes less accurate. In the ASO probe approach, by designing a fluorogenic probe for each individual tumor-specific MRD-PCR target, good sensitivities can be obtained (nearly 1.00 × 10^−5^). For MRD analysis, in fact, it is not only important to obtain quantitative data, but also to reach a sufficient sensitivity of the assay. The required sensitivity is, of course, dependent on the clinical application, but generally, the goal is to achieve a sensitivity of preferably 1.00 × 10^−4^/1.00 × 10^−5^; for example, sensitivities of at least 1.00 × 10^−4^ are needed to identify MRD-based low-risk ALL patients [5]. Moreover, when IG gene rearrangements are used as MRD-PCR targets, the sensitivity could be affected also by the ‘background’ of polyclonal lymphoid cells.

Actually, the introduction of qPCR for MRD detection in clinical trials has required the development of international guidelines and criteria for data analysis and laboratory reports, in particular to define sensitivity and quantitative range [5]. Moreover, quality control rounds are required to monitor the performance of the participating laboratories and to further improve and standardize qPCR analyses. For these purposes, the EuroMRD Consortium, founded in 2001 as a division of ESLHO (European Scientific foundation for Laboratory Hemato Oncology, see www.EuroMRD.org), developed common guidelines and quality control essential for development of standardization procedures in MRD analysis.

Different tissue samples can be analyzed in MRD monitoring assays; generally, bone marrow aspirate (BM) is considered the gold standard specimen for MRD detection in leukemia and in most lymphomas, due to the peculiar myelotropism of these disorders [2,3,26,27,28]. Moreover, in the rituximab era, the highly effective clearance of lymphoma cells ensured by the anti-CD20 monoclonal antibody rituximab suggested that BM might represent a more reliable specimen for MRD analysis, especially in FL [26]. Nonetheless, due to the higher availability and the minor discomfort for patients, peripheral blood (PB) and BM samples have been often used interchangeably, at least in FL and MCL, for molecular monitoring after achievement of clinical remission [26,27]. Regarding WM, there is ongoing debate whether to determine the presence of the MYD88^L265P^ mutation in sorted B-cells or in unselected BM mononuclear cells, while in PB samples the use of sorted B-cells over unselected mononuclear cells seemed to improve mutation detection rates [19,29].

### 1.4. Limitations of Standard PCR-Based MRD Techniques

Despite their continuous evolution since discovery, PCR methods still present some limitations.

One of the most relevant issues is the rate of marker identification failure: depending on the molecular target, the availability of consensus primers, the tumor infiltration of the analyzed tissue, as well as on the biology of the disease, a reliable MRD marker is found at diagnosis in only a portion of the investigated patients. In detail, a molecular marker based on BCL2/IGH is found in 55–60% of FL [2,28]; IGH rearrangements, instead, are not routinely employed in FL MRD analysis due to the relative instability of this kind of marker in FL setting. Similarly, an IGH rearrangement is found only in up to 50% of MM patients [30,31]. This is probably directly linked to the pathogenesis of MM andFL, arising from post-germinal center B cells, which have undergone, and, in FL, are subject to, ongoing somatic hypermutation [32]. On the other side, in MCL an IGH rearrangement is found in around 65–75% of patients, while BCL1/IGH *t* (11;14) translocation in about 30–40% [33,34,35].

Moreover, from a methodological point of view, labor intensiveness is a major concern, as well; the current ASO-PCR approach is characterized by the need to build a standard curve of progressive dilutions for the correct target quantification and to design patient-specific primers, in a non-automated process which requires laboratory personnel expertise and is indeed time-consuming [5]. 

Standardized techniques are needed to warrant robust and superimposable results among different laboratories, as the EuroMRD guidelines recommend [5]. False negative PCR results may be due either to a low sensitivity test, or to a low infiltration of disease in the specimen analyzed, or to clonal evolution patterns, which are not explored by routine analysis. In any case, misclassification of MRD results might expose patients to higher relapse risk as, inaccurately considered MRD negative, they are candidate to receive de-intensified treatment protocols.

In addition, interpretation of low-level qPCR results may be challenging: a critical limitation of qPCR is the inability to provide reliable target quantification for samples with a tumor burden between the sensitivity limit and the quantitative range of the method [5]; those samples are currently defined as “positive non-quantifiable” (PNQ). The occurrence of PNQs may be due to technical, assay-related issues, namely non-specific target amplification, resulting in a false positive result below the quantification threshold. Theoretically, PNQ results may also indicate a particular group of patients, with prognosis intermediate between MRD positive and negative, a population still not fully addressed by specific studies [36,37].

## 2. Digital PCR for MRD Monitoring

**Figure 1:**
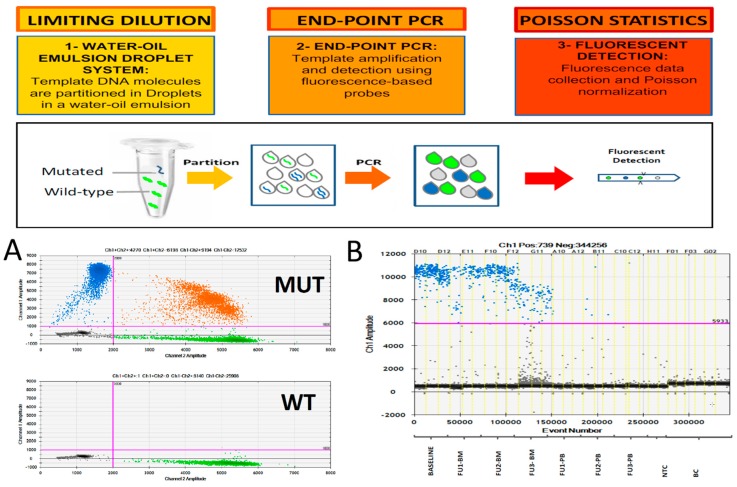
Droplet digital PCR key steps and workflow. (**A**) example of mutation detection assay; (**B**) example of immunoglobulin heavy chain minimal residual disease (IGH-MRD) monitoring (Baseline: diagnosis, FU: follow-up; BM: bone marrow; PB: peripheral blood, NTC: no template control, BC: buffy coat).

Recently, digital PCR (dPCR), one of the latest evolutions of PCR, has introduced several practical advantages to qPCR. dPCR was firstly described in 1999 [38,39], but only recently has the dPCR concept been translated into an easy and practical tool, and so far, there have been more than five hundred publications in the cancer field. Briefly, dPCR is based on tree compass points: (1) target compartmentalization, (2) end point PCR and (3) Poisson statistic (Figure 1).

Nowadays, five systems are commercially available, based on water-oil emulsion (BioRad-QX200 digital PCR System, Bio-Rad system, Hercules, CA, USA or JN Medsys-Clarity^®^ digital PCR system, JN Medsys, Singapore), micro-well chip-based (Life Technologies-QuantStudio3D^®^ Digital PCR, Life Technologies, Carlsbad, CA, USA) and microfluidic-chamber-based (Fluidigm-BioMark^®^HD, Fluidigm Corporation, San Francisco, CA, USA and Stilla Technologies-Naica Crystal dPCR, Stilla Technologies, Villejuif, France).

dPCR presents several practical advantages over qPCR, and might be particularly useful in the conduction of MRD studies. dPCR, not relying on a standard curve for sample quantification, can avoid the pitfalls associated with fluctuations in reaction efficiency, recovering those patients in which a standard curve could not be reliably generated, because of low tumor burden at diagnosis. Moreover, it can help in sparing precious diagnostic tissues, normally used for standard curve set up. Finally, several studies have reported the higher tolerance of dPCR to different types of inhibitors as compared to qPCR, thanks to the compartmentalization of target sequences in smaller volumes [40,41].

So far, only a few studies, afterwards described, report dPCR use for MRD monitoring in lymphoprolipherative disorders [23,42,43,44,45,46,47]. Actually, more progresses have been achieved by dPCR for MRD evaluation in other hematological malignancies. For instance, in chronic myeloid leukemia, dPCR has been largely shown to be more accurate at low transcript levels compared to standard qPCR [48,49,50,51,52].

In mature lymphoproliferative disorders, droplet dPCR has been shown to have a good concordance with qPCR [43]. The comparison of droplet dPCR (Bio-Rad system, Hercules, CA, USA) to the well-established qPCR-based method in MM, MCL, and FL indicates that droplet dPCR: (a) has sensitivity, accuracy and reproducibility comparable to qPCR; (b) shows an excellent correlation in all of the assessed disease entities and over a broad range of tumor infiltration rates [43]. Della Starza et al. confirmed these results in a study in which they monitored MRD levels of IGH rearrangements in ALL patients by both droplet dPCR and qPCR. In this study, results were concordant in 124/141 samples (88%) [44].

Recently, Cavalli et al. described a qPCR versus droplet dPCR comparison in paired PB and BM samples from FL patients [45]. BCL2 rearrangement was analyzed at diagnosis and after localized radiotherapy and rituximab administration, showing high overall concordance between droplet dPCR and qPCR (82%) and a higher sensitivity of droplet dPCR. Interestingly, droplet dPCR allowed to find a molecular marker in 44% of samples resulted negative by qualitative nested-PCR. Finally, tumor burden at diagnosis measured by droplet dPCR significantly predicted progression-free survival (PFS), compared to qPCR tumor quantification, which did not.

In a recent study, Guerrini F. et al. [46] used droplet dPCR for the detection of B-RAF^V600E^ mutation in HCL; MRD detection by this technique was significantly correlated to clinical status. Moreover, in this study, the authors compared the two quantitative methods showing a sensitivity of droplet dPCR more than half a log higher than qPCR, similar to the sensitivity levels reported for MYD88^L265P^ mutation detection; in fact, in WM patients, droplet dPCR reached a sensitivity of 5.0 × 10^−5^, compared to the 1.0 × 10^−3^ achievable by ASO-PCR [53]. Of note, all the above-mentioned studies used different criteria for positivity definition, indicating the need for common and standardized rules.

In summary, qPCR has been representing over time an innovative tool in molecular biology and clinical diagnostics; the spread of dPCR in the scientific community will also require time and validation by methodological standardization programs. Currently, multicenter efforts are ongoing, especially in the context of the EuroMRD group, to validate experimental set-up and standardized guidelines, in order to introduce droplet dPCR as routine MRD monitoring tool.

## 3. Next Generation Sequencing

Next generation sequencing (NGS) approaches have been widely used in many hematological diseases, to dissect the landscape of somatic mutations [54,55,56]. On the contrary, NGS methods for MRD analysis are still in their infancy and some issues, related to standardization, data interpretations and clinical impact, still need to be better defined [57,58,59].

NGS technology has been broadly used to overcome the previously mentioned disadvantages of MRD analysis using classical PCR methods. In particular, since 2010, most of the published NGS data have been generated using the LymphoSIGHT^®^ platform developed by Sequenta/Adaptive Inc. (San Francisco, CA, USA), a technology that targets the complete and incomplete B cell rearrangements by locus-specific multiplexed primers [58,60,61]. This NGS technique was originally tested on B-ALL samples, showing a higher sensitivity and feasibility when compared to the gold standard ASO-qPCR (sensitivity levels of 1.00 × 10^−6^ vs. 1.00 × 10^−5^, respectively [62]. Later, Ladetto M. confirmed the previous data and extended the NGS application to other hematological diseases such as MCL and MM, comparing the LymphoSIGHT^®^ platform with the well standardized IGH-based ASO-qPCR [57]. In this study, both tools were able to reach a sensitivity level of 1.00 × 10^−5^, and a good correlation of MRD results was observed (*R* = 0.791, *p* < 0.001), with superimposable target concordance in 80% of cases [57]. Moreover, few discordant cases, related to the presence of low level of bi-allelic rearrangements or to low tumor infiltration were detected [57]; in these cases, NGS was able to recognize molecular markers with an MRD profile completely different from those identified and monitored by qPCR.

Based on these evidences, several studies were performed to define the clinical impact of LymphoSIGHT^®^ NGS in ALL, MCL, CLL and MM for MRD analysis. Experiments were also carried out in the context of different clinical trials, with the aim to assess the outcome predictor value of this technology [58,60,61,63,64,65,66,67,68,69,70,71,72,73,74]. In particular, Martinez-Lopez et al. assessed that NGS method in MM has applicability almost comparable to MFC, but is able to reach higher sensitivity, with MRD level by NGS correlating with time to progression and OS [58].

**Figure 2:**
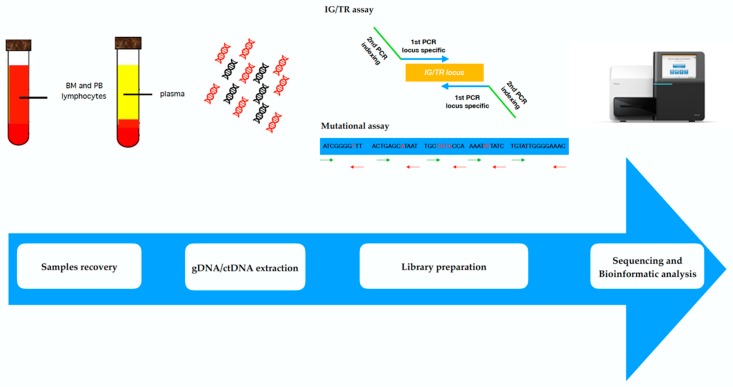
Next generation sequencing workflow. BM: bone marrow; PB: peripheral blood; ctDNA: circulating tumor DNA; IG/TR: immunoglobulin/T cell receptor.

Although the LymphoSIGHT platform has been shown to be a feasible and predictive MRD approach, there are many aspects that still need to be defined in order to make NGS approach more user-friendly in routine clinical practice [59,75]. This aim is currently an important goal of the Euro-MRD [5] within the EuroClonality NGS group (EC-NGS, see www.EuroClonalityNGS.org), involving several NGS experienced groups working on the standardization of IGH assay design and bioinformatics analysis [76,77,78,79]. Based on this need, an academic-based, reproducible approach for IGH-based MRD study has been developed (Figure 2). Preliminary data from the first pilot studies, performed on ALL samples, have shown intra and inter-laboratory concordant results, using two different platforms (Illumina, San Diego, CA, USA and ThermoFisher, Cambridge, MA, USA).

The EC-NGS approach proved to be a robust, feasible tool for MRD monitoring and early relapse prediction in ALL patients [79]. In particular, it was able to assign to a different category those patients that achieved complete remission but scored as PNQ by qPCR during post-transplant follow-up. When analyzed by deep sequencing, these patients resulted as false positives and were therefore re-defined as MRD negative, better correlating with clinical outcome. In the same study, a second cohort of patients with low positivity qPCR results, who relapsed after stem cell transplantation, were confirmed to be MRD positive by NGS. From these data, it was possible to assume that the low-level positivity detected by qPCR in the first group might refer to physiological B rearrangements, revealing NGS as a reliable and more accurate method for MRD monitoring [36].

Moreover, the EC-NGS assay has been preliminary tested in FL to implement IGH clonal rearrangements as molecular marker, since IGH MRD by qPCR is less feasible in this disease, due to the impact of ongoing somatic hypermutation [80]. Presently, this is the only study showing that NGS-MRD is able to identify an IGH clonotype marker in around 60% of FL patients, and highlighted the MRD positivity impact on clinical outcome.

Finally, high throughput technologies like deep sequencing are able to generate massive amount of biologic information. Annotation and interpretation of the millions of nucleotides defining the IG sequences require ad hoc developed bioinformatics software. For these purposes, several web-based, open source tools were designed for the analysis of IG deep sequencing data, aiming at disease clonality identification, with different characteristics in terms of flexibility, user-friendly features, interactivity, customization [78,81,82,83,84,85]. Continuous updating of this software is required in order to chase after the newly developed applications of NGS tools, making the connection between bioinformatics and health technology innovation more and more crucial [86]. Due to the algorithm complexity, these tools have not yet been systematically compared, therefore the analyses reproducibility among them is still a debated issue.

## 4. Liquid Biopsies: Novel Techniques for ctDNA Detection in Lymphoproliferative Malignancies

An appealing new frontier for MRD development in lymphoma is the so called “liquid biopsy”, that is, the detection of circulating tumor DNA (ctDNA) on plasma or other biological fluids. In fact, ctDNA can be found in almost all available biologic fluids, the most frequently employed being blood and urine, but it is also evaluable in cerebrospinal fluid, stool and pleural effusion [87].

Briefly, the advantages of liquid biopsy compared to PB or BM samples are related: (1) to the widening of non-invasive MRD and mutational studies also to non-leukemic diseases (such as aggressive lymphomas, plasma cell disorders, as well as solid tumors); (2) to the peculiarity of representing the heterogeneity of tumor tissues, rather than targeted surgical biopsies [88]; (3) to the non-invasive, “patients-friendly” collection procedure, leading to the possibility of increasing the time points of samples collection during follow-up [87,89].

However, until recently, the available molecular techniques did not achieve a sufficient sensitivity to be able to detect a small amount of ctDNA. The recent advances in PCR and sequencing tools have made it possible to apply the liquid biopsy also to hematologic diseases; thus, ctDNA and dPCR analyses for lymphoproliferative diseases could be a promising option in order to determine the initial genomic profile, monitor treatment response and eventually to assess the emergence of new mutations leading to therapy resistance mechanisms.

In his proof-of-concept study, Camus V. demonstrated the feasibility, simplicity and reproducibility of dPCR to detect and quantify somatic mutations in ctDNA extracted from DLBCL patients’ plasma [47]. In addition, Camus V. also demonstrated that classical HL patients with XPO1 mutations, detected by dPCR in cfDNA from plasma collected at the end of standard chemotherapy, had a shorter PFS compared to unmutated XPO1 cases [23]. These results suggest that the clearance of the XPO1 mutation in plasma may represent a new prognostic marker, although the initial findings are preliminary and retrospective.

In MM, this approach could have several potential applications, since recent findings indicate that monoclonal (M) component and ctDNA may be independently released in the bloodstream. Interestingly, MM monitoring by ctDNA may be thus possible in patients who cannot be reliably followed up through M component variations, and recurrent mutation analysis by dPCR and NGS may be a potential driver to targeted therapy [90].

The first steps to investigate the feasibility of an MRD test on ctDNA in the setting of WM have been also made, and a recent study showed good correlation in MYD88^L265P^ mutation detection between plasmatic and BM compartments and superior detection rate in plasma compared to PB [53].

Furthermore, since the imaging methods currently employed for response assessment in DLBCL patients are not able to capture the dynamic process of response kinetics and/or treatment resistance [91], the combination of NGS and ctDNA could allow a more precise risk stratification and outcome prediction [92]. Several groups focused their studies to assess the clinical impact of MRD monitored by NGS in liquid biopsy. In fact, ctDNA analysis for MRD purposes was a powerful outcome prediction tool in lymphoma patients [68,93,94]. In particular, in DLBCL patients, surveillance monitoring of IGH-based clonotypes on ctDNA after the achievement of complete remission demonstrated an advantage in PFS for MRD negative patients, and was able to predict disease recurrence before its clinical evidence [95]. Moreover, in a proof-of-concept study, tumor-specific clonotypes were identified by NGS in 8/11 HL patients [96]. NGS sequencing of the IG VDJ locus on plasma samples was also employed in FL, refining tumor subclone dissemination and confirming both the ability of ctDNA to reflect clonal heterogeneity and its prognostic role on PFS [97].

A different approach for liquid biopsy is to identify and simultaneously track by NGS recurrent somatic mutations, rather than focusing on IG clonotypes.

In a study by Fontanilles et al., preliminary data from 25 primary central nervous system lymphoma (PCNSL) patients were obtained comparing results of targeted sequencing of ctDNA and genomic tumor DNA. Tumor volume or location in deep brain structures did not affect somatic mutations discovery in plasma, although the assay lacked sensitivity, possibly due to intrinsic limits of the adopted NGS method [98].

In fact, to improve mutational screening and correlation with outcome, novel NGS tools, targeting recurrently mutated genes have been designed. Cancer Personalized Profiling (CAPP) sequencing, an ultrasensitive sequencing method, was first implemented in solid tumors, in particular NSCLC [99]. This high throughput method allows high sensitivity analysis, despite being more complex than IGH-based monitoring. Scherer F. applied CAPP-sequencing to tumor biopsies and ctDNA of 92 DLBCL patients: ctDNA level strongly correlated with clinical prognostic indexes, and interestingly, it was also able to determine cell of origin and risk of aggressive transformation in indolent lymphomas [100].

In a subsequent study on a larger series, ctDNA was detectable prior to treatment in 97% of patients, and its levels differed significantly according to prognostic risk group and stage as previously described; in addition, a good correlation between metabolic tumor volume (MTV) by PET scan and ctDNA level was described [101]. More importantly, ctDNA load was also associated with event-free survival (EFS) and OS [101]. Finally, CAPP sequencing was also used to discover and track lymphoma-associated mutations, with a sensitivity higher than 90% and specificity near to 100%, mirroring tissue biopsy, while better representing spatial tumor heterogeneity. After conventional therapy, rapid clearance of DLBCL mutations was observed in plasma samples of responding patients, while in resistant cases the basal mutational pattern was still present, seldom with additionally acquired mutations selected by clonal evolution [25]. Similarly, in HL patients, the treatment-dependent modifications in ctDNA were monitored by CAPP sequencing, identifying clonal evolution patterns; ctDNA was also able to identify false positive and false negative cases by PET, which would be particularly useful for patients treated with checkpoint inhibitors, for whom response monitoring by imaging techniques can be challenging [102].

Nonetheless, a strict standardization of laboratory procedures, interpretation guidelines and data validation are needed to introduce ctDNA analysis in clinical practice. In particular, pre-analytic collection and processing steps will require validation. In fact, delay in sample processing and temperature of blood samples before centrifugation can affect ctDNA retrieval. Indeed, all the aforementioned studies applied liquid biopsy techniques in a mono-centric setting, allowing for short patient-to-bench time and reduction of these pre-analytic biases. When multi-center trials with liquid biopsies were first designed, this issue was faced with the adoption of specific “BCT” tubes (Streck), able to overcome the need for rapid sample processing by reducing DNA degradation, like in many ongoing trials (NCT02371148, NCT02390869, NCT02710643, NCT02858258, NCT03521596, EudraCT 2017-004628-31) [103,104,105,106].

## 5. Concluding Remarks

This review describes the latest molecular technologies introduced for MRD monitoring in B cells lymphoproliferative diseases, dPCR and NGS. These new methods show super-imposable results to the gold standard qPCR, suggesting a promising role for technical improvement and widening of clinical applications.

A schematic comparison between dPCR and NGS for MRD monitoring and mutation detection is provided in Table 1.

Droplet dPCR has been shown as accurate and sensitive approach, able to overcome some qPCR drawbacks in sensitivity, feasibility and reproducibility, thus representing an attractive alternative for MRD analysis in many hematologic diseases.

NGS is a new, powerful tool, that has already enlighted the mutational landscape of many subtypes of lymphoma, improving the knowledge of the clonal heterogeneity and of the kinetics of these diseases.

Actually, both these techniques appear highly sensitive, accurate and reproducible; interestingly, they share the possibility to be applied on easily obtainable tissue samples, removing the need to perform surgical biopsies or tight radiological monitoring. Definitely, NGS introduction could change the quality of routine laboratory diagnosis, speeding up the analysis of a huge number of samples. Nevertheless, its cost and not-yet-standardized approach for MRD monitoring is still an open issue. Droplet dPCR, on the contrary, is a simple tool, with a fast turn-around time and lower costs than NGS, but it can identify only known targets, following no more than one marker at the same time. Probably, especially for the purpose of liquid biopsies, the right solution would be to introduce NGS as a marker screening method, able to highlight a plethora of new molecular targets, and to further validate and investigate them with dPCR, ideal for molecular monitoring.

While not being the focus of this review, in recent years also, MFC has increased its sensitivity and reliability for MRD monitoring; the Next Generation Flow, that relies on the simultaneous assessment of ≥8 parameters, leads to identification of aberrant cells with a detection limit of 1.00 × 10^−5^ [107,108]. Possible drawbacks of this technique are the need for high laboratory expertise and the lack of standardization, even though the EuroFlow Consortium (see www.EuroFlow.org) is progressing in the development of standardized, accurate MFC protocols.

Undoubtedly, the clinical feasibility of molecular MRD assessment by dPCR and NGS is still in its infancy; in particular, prognostic significance of the newly developed techniques needs to be determined. In fact, so far, few observations have reported an impact of different molecular tools on clinical outcomes; however, many of the studies published were performed on relatively small series of patients, and in some cases in retrospective analyses. Most of the evidence sustaining the correlation of new molecular tools with patients’ outcome derives from liquid biopsy applications, particularly for DLBCL, MM and FL [9,36,96,102,103].

In conclusion, the studies published so far show the high potential of dPCR and NGS to change the current molecular MRD monitoring approach, allowing in future trials a better characterization of hematologic diseases and leading to a true MRD-driven, personalized approach.

## Figures and Tables

**Table 1:** Technical comparison of droplet dPCR and next generation sequencing (NGS) for MRD study in lymphoproliferative diseases.

Technique	Approach	Sensitivity	Advantages	Disadvantages
Droplet digital PCR	Targeted analysis	Up to 5.00 × 10^−5^ [23,47,53]	Absolute quantification of target (no need of standard curve)	Discovery of unknown mutations not possible
			High precision measure even at low concentration targets	Not able to overcome the limitation of allele-specific design
NGS	(a) Multiple targets; (b) Whole genome; (c) Whole exome	Up to 1.00 × 10^−4^ [25,101]	Potentially highly sensitive	Long turn-around time, not fully standardized
			Allows discovery approach	Bionformatic tools and expert personnel needed
			No need for patient-specific reagents	Expensive
